# Supplementary material for: Photon-directed multiplexed enzymatic DNA synthesis for molecular digital data storage
Source: Nat Commun. 2020 Oct 16;11:5246. doi: 10.1038/s41467-020-18681-5 (PMC7567835; doi:10.1038/s41467-020-18681-5)
Supplement: Supplementary file 1 — Supplementary Information [file 41467_2020_18681_MOESM1_ESM.pdf]

Supplementary Information

Photon-directed Multiplexed Enzymatic DNA Synthesis for Molecular Digital Data Storage

Authors: Howon Lee[1,2], Daniel J. Wiegand[1,2], Kettner Griswold[1,2,3,4], Sukanya Punthambaker[1,2], Honggu Chun[5], Richie E. Kohman[1,2,*], George M. Church[1,2,*]

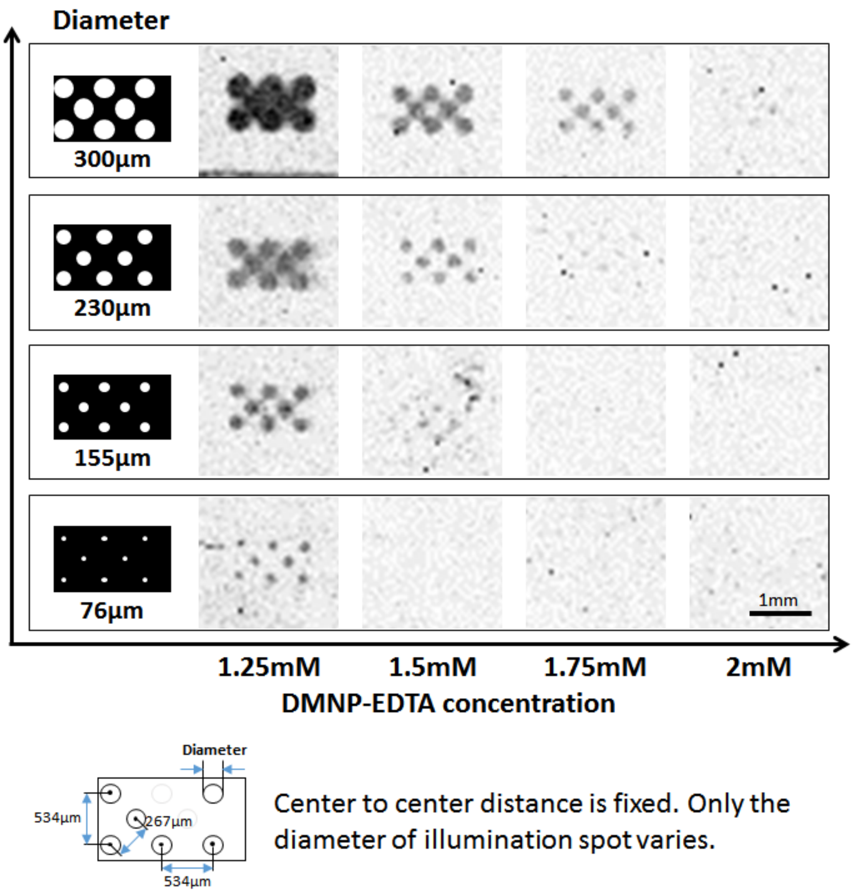

Supplementary Figure 1: A comparison of DMD pattern fill factor and concentration of the caging molecule, DMNP-EDTA. The fill factor is defined as the ratio between the illuminated and surrounding area within the pattern. For circular spots, the fill factor is dictated by their diameter. A finely tuned balance between a pattern's fill factor and caging molecule concentration present in the reaction master mix is required for well-confined nucleotide extension. This is indicated by sharp patterning and no cross-talk between the individual spots post-synthesis. As the fill factor (diameter of the spots) increases, less DMNP-EDTA is present in the surrounding area to chelate free Co^{2+} that diffuses away from the illuminated pattern. Increasing the total concentration of DMNP-EDTA will help eliminate significant cross-talk between spots; however, too much DMNP-EDTA will quench synthesis reactions before visible oligonucleotide extension can occur. Balanced fill factor and DMNP-EDTA is dependent on the total concentration of Co^{2+} initially supplemented in the synthesis master mix. Optimization took place with 1 mM initial Co^{2+}, 8 seconds of UV irradiation, and 15 seconds of post-illumination incubation for all conditions. Images were captured and analyzed using a Typhoon FLA 9000 Imager. The y-axis indicates the diameter of the circular spots and masks used to generate the illumination pattern. The x-axis indicates the total concentration DMNP-EDTA in the reaction mixture. Additionally, a diagram showing the center-to-center vertical, diagonal and horizontal distances between spots are provided.

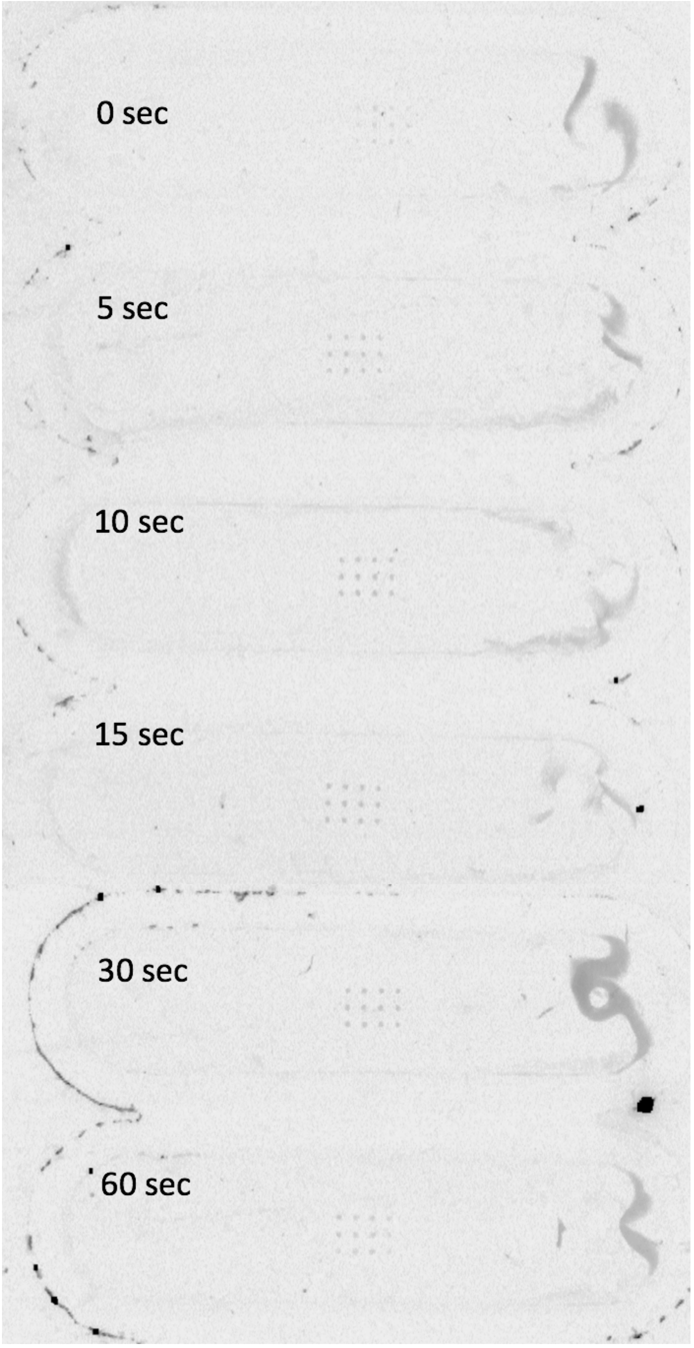

Supplementary Figure 2: A comparison post-illumination incubation times. It is essential to allow sufficient time for TdT to incorporate nucleotides onto the surface bound oligonucleotide before washing away the enzyme master mix containing free Co^{2+} released from photolabile DMNP-EDTA upon UV irradiation. Using sequence-specific splint-end ligation with short visualization pro containing a 3'-Cy3 fluorophore, it was found that at least 5 seconds of post-illumination incubation is required for high-quality surface extension. Since DMNP-EDTA is supplemented in the enzyme master mix in excess, there were no significant differences between post-illumination incubation times longer than 15 seconds.

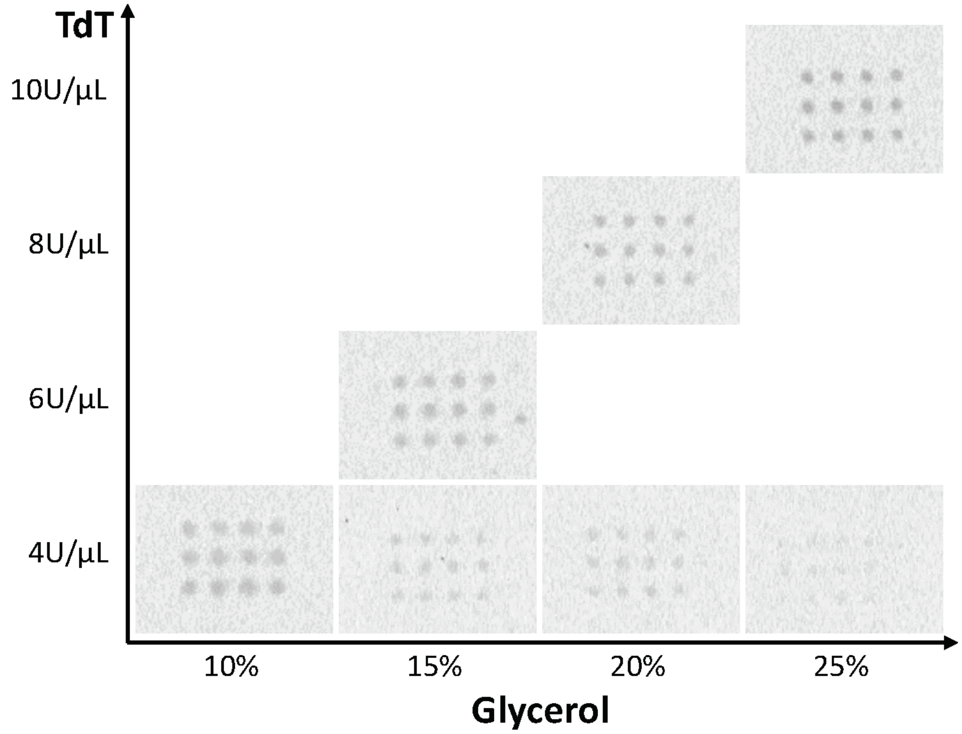

Supplementary Figure 3: A comparison of glycerol percentage and concentration of TdT in the synthesis master mix. Increasing the glycerol percentage leads to slower enzyme kinetics, but can be overcome by increasing the total concentration of TdT. The concentration of TdT is defined in units per volume. One unit of TdT catalyzes the incorporation of 1 nmol of deoxythymidylate into a polynucleotide fraction in 60 min at 37 °C (Thermo). However, all reactions were performed at room temperature.

S4a

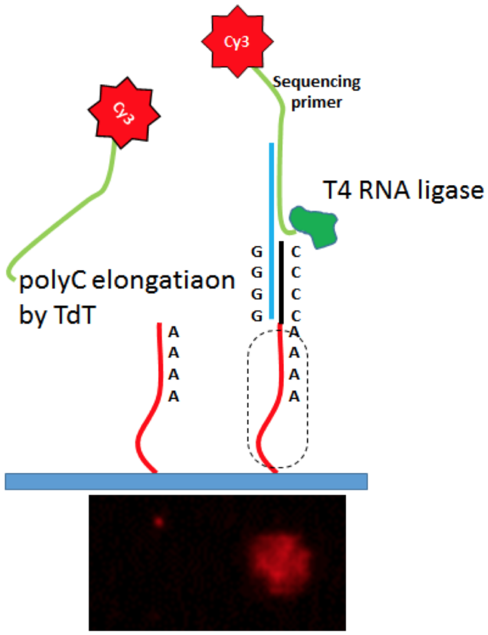

S4b

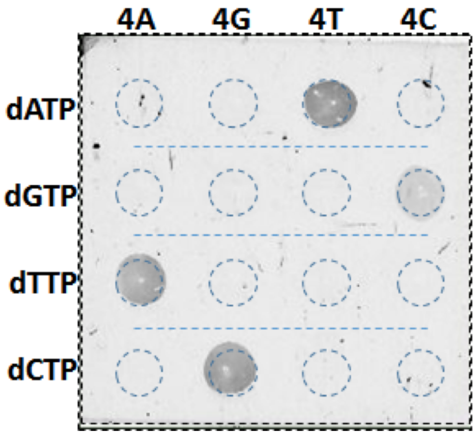

Supplementary Figure 4: a, Specific splint-end ligation mechanism for visualization of oligonucleotide synthesis. Surface bound oligonucleotides first undergo a final 3'- extension using the "C" nucleotide by TdT. A ligation master mix containing T4 RNA Ligase, a splint oligonucleotide, and a 3'-Cy3 labeled probe is then incubated with the surface-bound oligonucleotide to attach the probe. After thorough washing, surface bound oligonucleotides can be visualized with fluorescence imaging. An extension of a minimum 4 nucleotides is required for probe attachment using the splint oligonucleotide. Ligation of PCR or NGS adaptors can be attached to surface-bound oligonucleotide in the same manner. **b,** Final extension can be performed with any of the natural nucleotide bases and is not restricted to "C". For example, a final extension with dATP, requires a splint with a set of "T". This is demonstrated for each nucleotide extension and splint oligonucleotide combination. Dark spots indicate successful ligation of Cy3 probe.

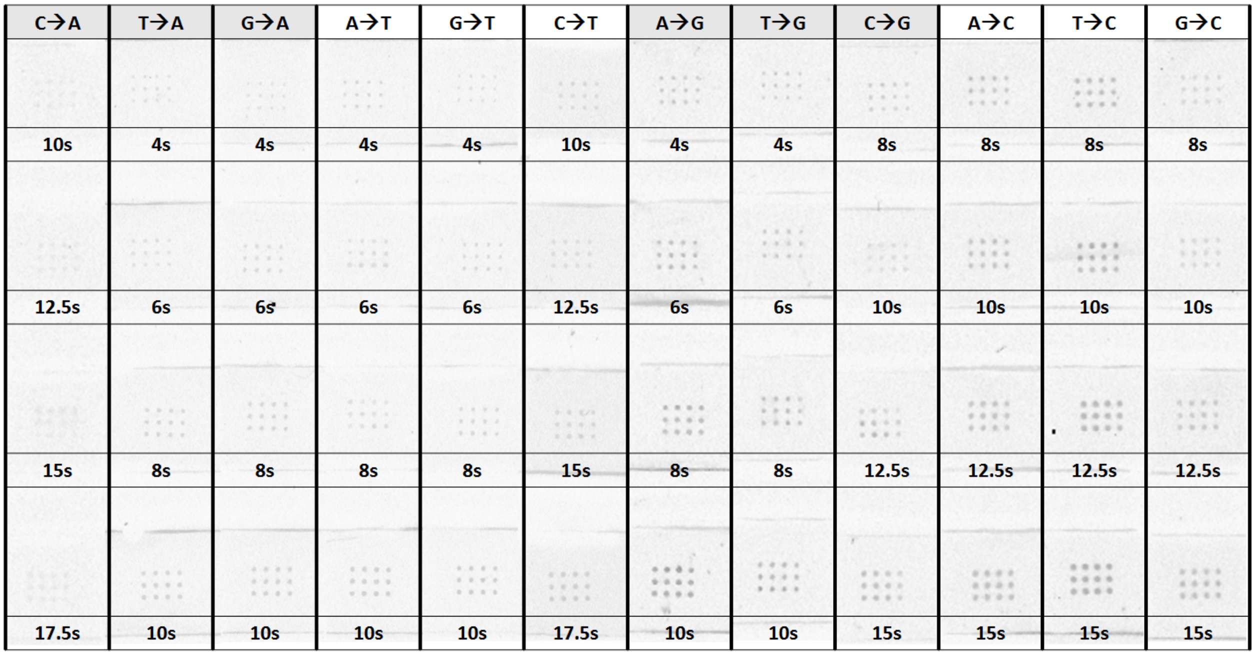

Supplementary Figure 5: Verification of the base addition time. The base addition time for each possible base transition was empirically determined by testing four discrete illumination time followed by fluorescence imaging via splint-end ligation of a probe sequence containing a 3'-Cy3 fluorophore. We chose the base addition time of each case based on the fluorescence signal intensity and spot confinement at the same time. As expected, most "C" involving additions tend to require more illumination time than others.

Index	Template
0	GAGACACT
1	GAGTATAG
2	GAGCATGA
3	GACTATAG
4	GACGCATC
5	GACACGAG
6	GATCGCGA
7	GATATGCT
8	GATGCTAG
9	GTCTGCAT
10	GTCGATAG
11	GTCATGCA

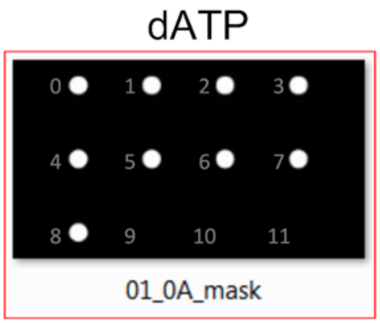

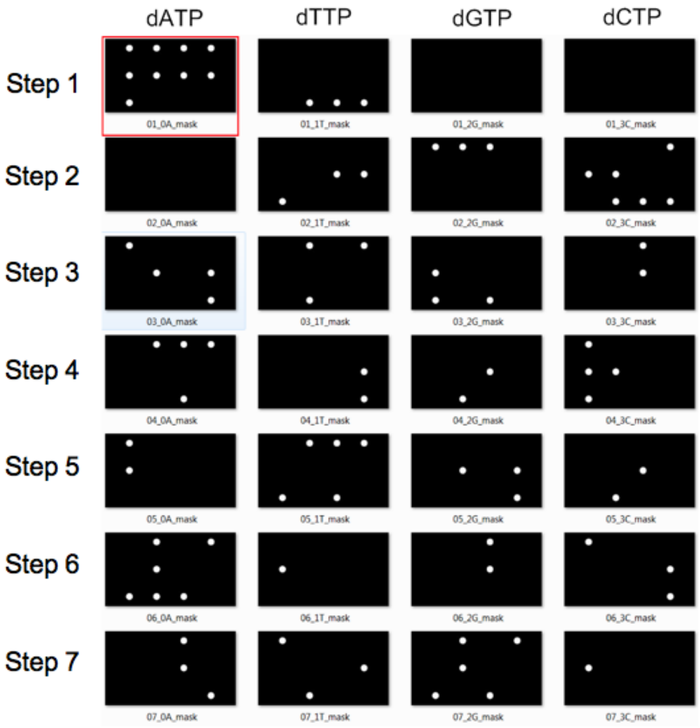

Supplementary Figure 6: An overview of all 29 dynamic masks needed to optimally synthesize the 12 DNA oligonucleotide sequences encoding the simplified "Overworld Theme" melody using a (3 × 4) pattern with 100 µm circular spots in multiplex. Each row of masks represents a single synthesis step, which encompasses the delivery of each natural nucleotide and the illumination pattern necessary for their spatially specific incorporation as individual cycles. For example, in the synthesis step 1, the "A" nucleotide is needed for sequences being synthesized at indices 0 through 8. This is followed by the "T" nucleotide, which is needed at indices 9 through 11. The "G" and "C" nucleotides are not needed so no masks are generated and the DMD does not illuminate any of the spots during synthesis step 1. This process can be altered to accommodate the synthesis of more sequences, nucleotide bases, and the total sequence template length. On Step 8, all spots on the array are illuminated using one mask to perform the final "C" extension (not shown).

S7a

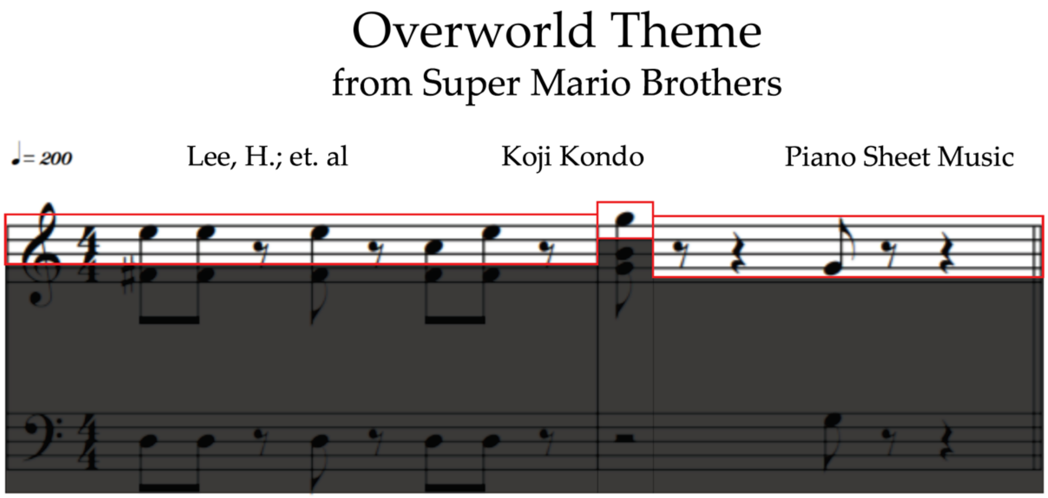

S7b

Index	0	1	2	3	4	5	6	7	8	9	10	11
Note	E5	E5	G#$_0$	E5	G#$_0$	C6	E6	G#$_0$	G6	G#$_0$	G5	G#$_0$
Tempo	♪	♪	𝄾	♪	𝄾	♪	♪	𝄾	♪	𝄾𝄽	♪	𝄾𝄽

Supplementary Figure 7: a, Piano sheet music showing the first two measures of the "Overworld Theme" composed by Koji Kondo for the 1985 Nintendo Entertainment System (NES) video game Super Mario Brothers. **b,** Musical notes and their tempos were extracted and indexed from the sheet music to produce a simplified melody to be encoded into DNA sequences as indicated in the table. Rests, where no musical note is played in the melody, were assigned to the note G#$_0$, which plays at a frequency that is inaudible to the ordinary adult human [2]. The simplified melody is represented on the sheet music with the red box. Grayed-out musical information was not encoded in DNA.

S8a

Original Midi Chart

Octave	Note Numbers											
	C	C#	D	D#	E	F	F#	G	G#	A	A#	B
-1	0	1	2	3	4	5	6	7	8	9	10	11
0	12	13	14	15	16	17	18	19	20	21	22	23
1	24	25	26	27	28	29	30	31	32	33	34	35
2	36	37	38	39	40	41	42	43	44	45	46	47
3	48	49	50	51	52	53	54	55	56	57	58	59
4	**60**	**61**	**62**	**63**	**64**	**65**	**66**	**67**	**68**	**69**	**70**	**71**
5	**72**	**73**	**74**	**75**	**76**	**77**	**78**	**79**	**80**	**81**	**82**	**83**
6	**84**	**85**	86	87	88	89	90	91	92	93	94	95
7	96	97	98	99	100	101	102	103	104	105	106	107
8	108	109	110	111	112	113	114	115	116	117	118	119
9	120	121	122	123	124	125	126	127				

Subtract default note number (60)

Modified Midi Chart

Octave	Note Numbers											
	C	C#	D	D#	E	F	F#	G	G#	A	A#	B
-1	-60	-59	-58	-57	-56	-55	-54	-53	-52	-51	-50	-49
0	-48	-47	-46	-45	-44	-43	-42	-41	-40	-39	-38	-37
1	-36	-35	-34	-33	-32	-31	-30	-29	-28	-27	-26	-25
2	-24	-23	-22	-21	-20	-19	-18	-17	-16	-15	-14	-13
3	-12	-11	-10	-9	-8	-7	-6	-5	-4	-3	-2	-1
4	**0**	**1**	**2**	**3**	**4**	**5**	**6**	**7**	**8**	**9**	**10**	**11**
5	**12**	**13**	**14**	**15**	**16**	**17**	**18**	**19**	**20**	**21**	**22**	**23**
6	**24**	**25**	26	27	28	29	30	31	32	33	34	35
7	36	37	38	39	40	41	42	43	44	45	46	47
8	48	49	50	51	52	53	54	55	56	57	58	59
9	60	61	62	63	64	65	66	67	-60	-60	-60	-60

S8b

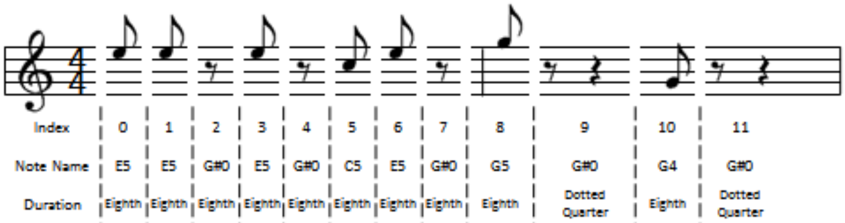

Duration		Decimal
𝄾	♪	0
𝄽	♩	1
𝄽.	♩.	2

S8c

Index (0/11) Ternary form	Note name	Note number (# - default octave 60)	Note number (Ternary form)	Duration	Ternary data	Template Sequence (G start)
000	E5	76 (16)	121	0 (1X eighth note)	0001210	GAGACACT

Supplementary Figure 8: a, The indexed notes from the simplified melody are assigned a note number based on a modified Musical Instrument Digital Information (MIDI) note chart, which indicates both the note and the octave at which it is played at. **b,** These numbers are converted into ternary and combined with the ternary forms of the index and duration number assignments for the note, yielding a 7 digit sequence of numbers. **c,** For example, the first note in the simplified melody is E5 with a quarter note duration. This yields the ternary data **0001210**, where **000** indicates that it is the first note to be played, **121** indicates that the note is E at the 5th octave, and **0** indicates that it should be played for the duration of one eighth note. From this, a template DNA sequence is mapped using previously described methods [1].

Source for MIDI conversion in MatLab:
https://www.mathworks.com/help/audio/ref/midimsg.html

Index	Note name	Note number	Note # -default octave	Duration	Ternary Index	Ternary Note number	Ternary Duration	Ternary Data	Template
0	E5	76	16	Eighth	000	121	0	0001210	GAGACACT
1	E5	76	16	Eighth	001	121	0	0011210	GAGTATAG
2	G#0	20 (86)	26	Eighth	002	222	0	0022220	GAGCATGA
3	E5	76	16	Eighth	010	121	0	0101210	GACTATAG
4	G#0	20 (86)	26	Eighth	011	222	0	0112220	GACGCATC
5	C5	72	12	Eighth	012	110	0	0121100	GACACGAG
6	E5	76	16	Eighth	020	121	0	0201210	GATCGCGA
7	G#0	20 (86)	26	Eighth	021	222	0	0212220	GATATGCT
8	G5	79	19	Eighth	022	201	0	0222010	GATGCTAG
9	G#0	20 (86)	26	Eighth+Quarter	100	222	2	1002222	GTCTGCAT
10	G4	67	7	Eighth	101	021	0	1010210	GTCGATAG
11	G#0	20 (86)	26	Eighth+Quarter	102	222	2	1022222	GTCATGCA

Supplementary Figure 9: A table outlining the fully converted "Overworld Theme" simplified musical melody to ternary information and then mapped to a DNA template sequence to be synthesized in multiplex. Note that all mapped DNA template sequences start with the "G" nucleobase, this is the 3'- end of the surface-bound initiator oligonucleotide that all sequences will be synthesized from.

S10a

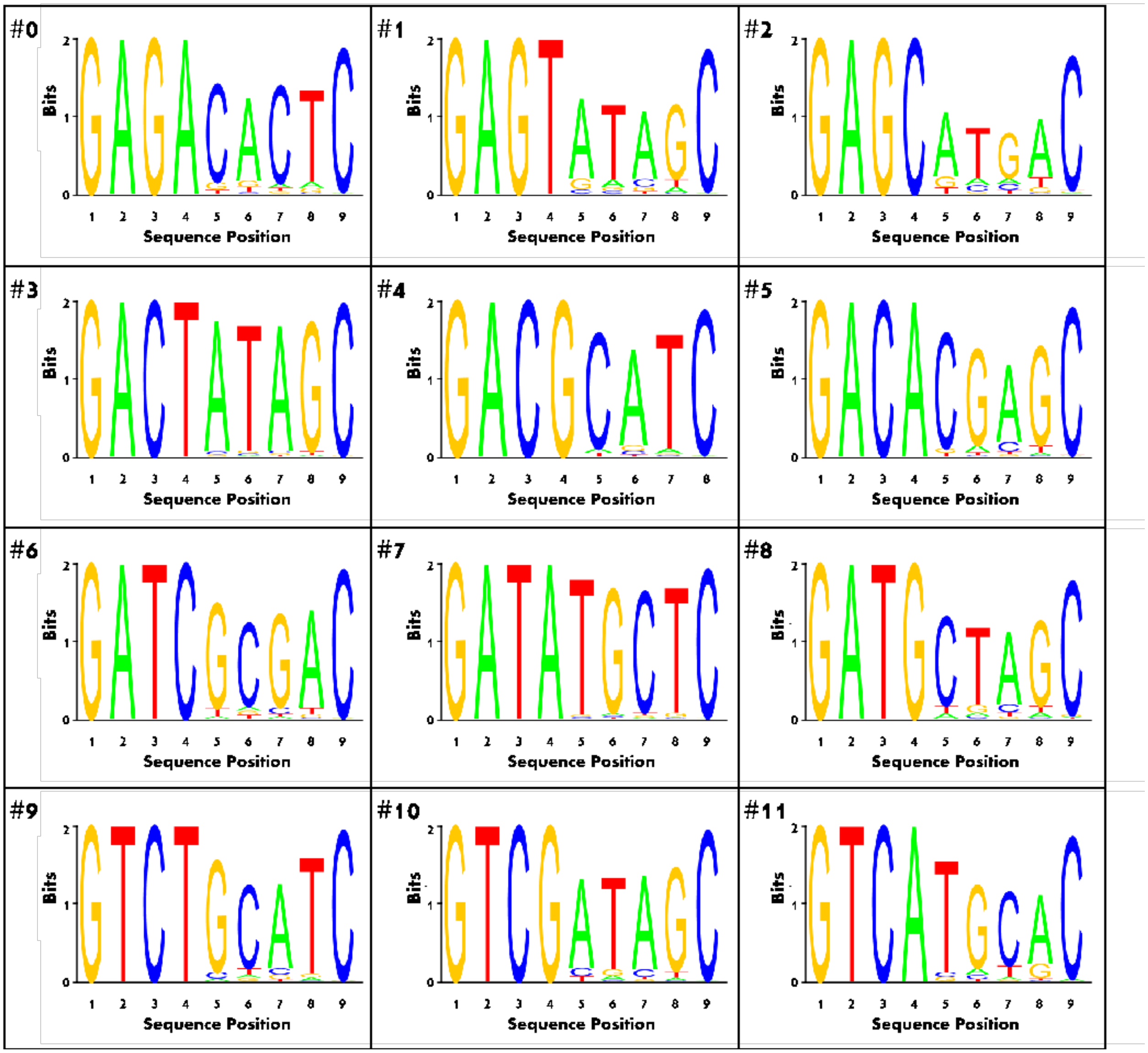

S10b

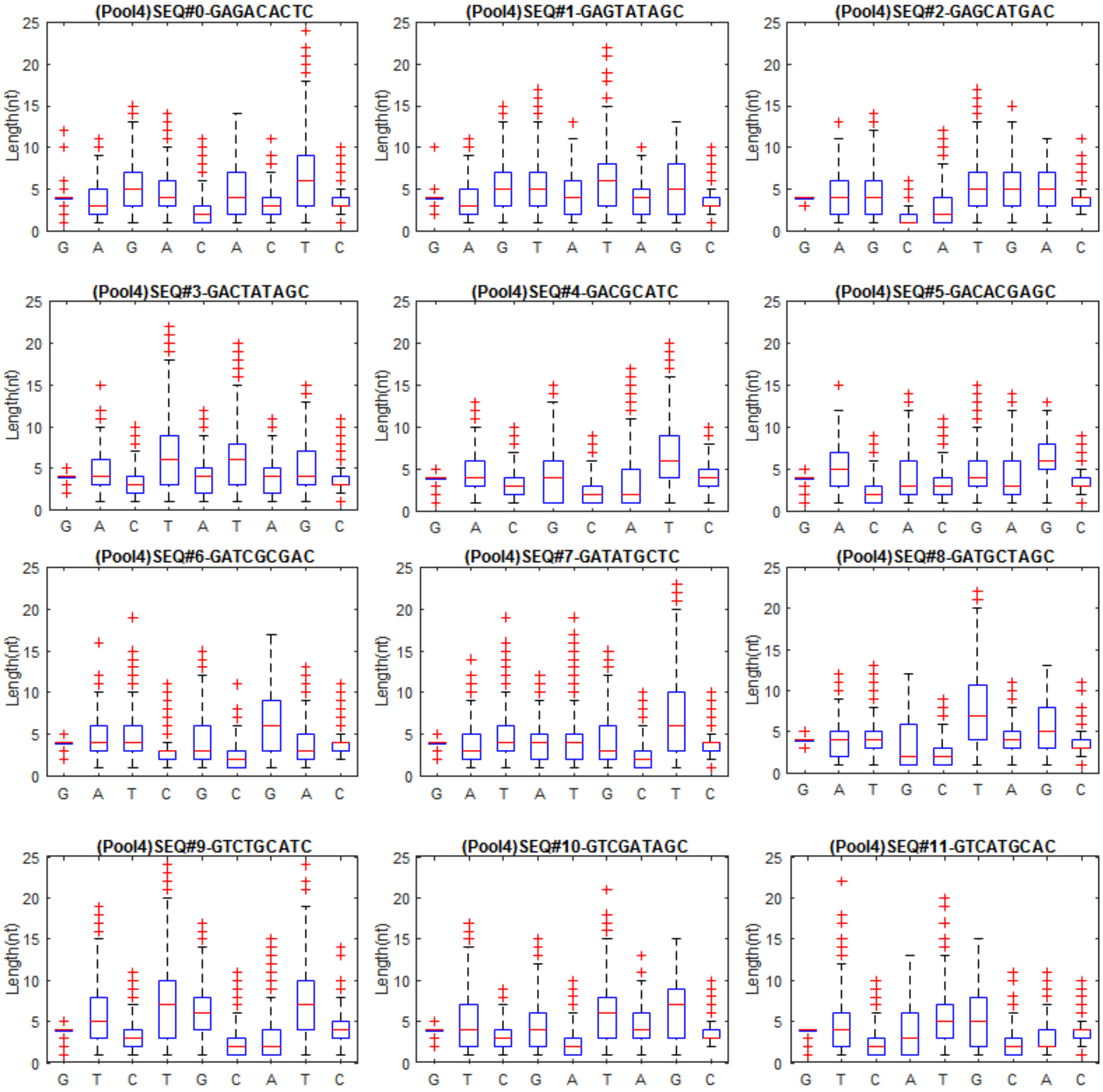

(Pool4)SEQ#0-GAGACACTC
(Pool4)SEQ#1-GAGTATAGC
(Pool4)SEQ#2-GAGCATGAC
(Pool4)SEQ#3-GACTATAGC
(Pool4)SEQ#4-GACGCATC
(Pool4)SEQ#5-GACACGAGC
(Pool4)SEQ#6-GATCGCGAC
(Pool4)SEQ#7-GATATGCTC
(Pool4)SEQ#8-GATGCTAGC
(Pool4)SEQ#9-GTCTGCATC
(Pool4)SEQ#10-GTCGATAGC
(Pool4)SEQ#11-GTCATGCAC
Length(nt)
0 5 10 15 20 25
G A G A C A C T C
G A G T A T A G C
G A G C A T G A C
G A C T A T A G C
G A C G C A T C
G A C A C G A G C
G A T C G C G A C
G A T A T G C T C
G A T G C T A G C
G T C T G C A T C
G T C G A T A G C
G T C A T G C A C

S10c

**Total reads**	**216252**
**Sequence with adaptor**	**100760**
**Perfect transition match**	**14335**

Sequence	GAGACACTC	GAGTATAGC	GAGCATGAC	GACTATAGC	GACGCATC	GACACGAGC	GATCGCGAC	GATATGCTC	GATGCTAGC	GTCTGCATC	GTCGATAGC	GTCATGCAC
Sequence index	0	1	2	3	4	5	6	7	8	9	10	11
Filter 1,2,3 (# of transition, index)	1742	605	426	2357	2268	1053	1182	2668	536	2344	878	682
Filter 4 (perfect match)	1335	466	303	2182	2014	868	996	2492	423	1993	739	524

Supplementary Figure 10: In-depth analysis for Illumina MiSeq sequencing. **a,** Sequence logo representation of all 12 strands after *in silico* filter 1, 2, and 3. **b,** Box plot statistical information for the extension length distribution for each base transition for all perfectly matched (*in silico* filter 1, 2, 3, and 4) 12 oligonucleotides synthesized in multiplex. Red pluses on box-plots represent statistical outliers. Each SEQ represents an individual subset/index and DNA oligonucleotide. **c,** Raw sequencing data was filtered as per specified elsewhere for perfect matches containing all 8 base transitions for each oligonucleotide index. The breakdown for filtering from the raw sequencing data is included.

Source for SeqLogo in MatLab:
https://www.mathworks.com/help/bioinfo/ref/seqlogo.html

S11a

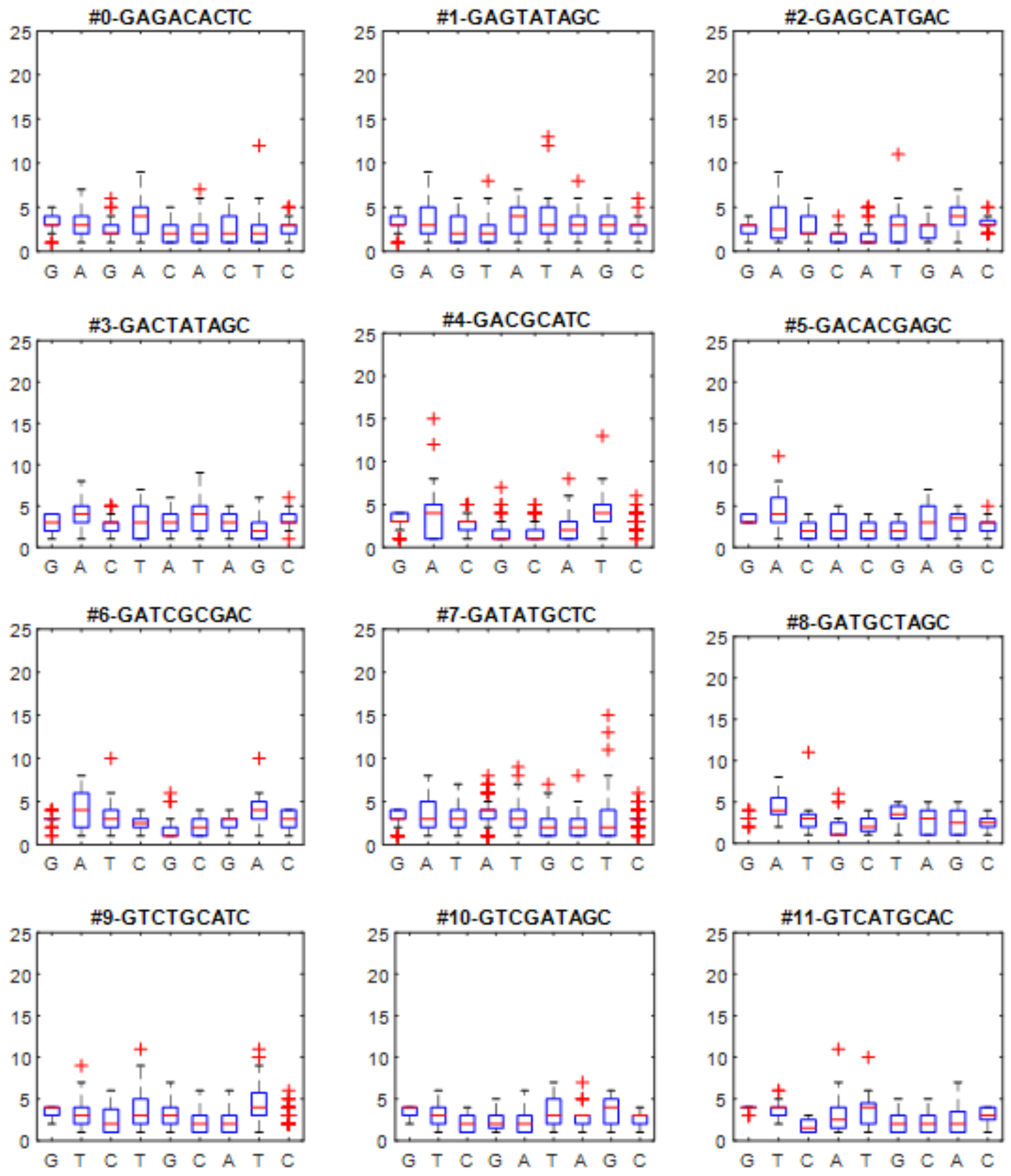
#0-GAGACACTC
#1-GAGTATAGC
#2-GAGCATGAC
#3-GACTATAGC
#4-GACGCATC
#5-GACACGAGC
#6-GATCGCGAC
#7-GATATGCTC
#8-GATGCTAGC
#9-GTCTGCATC
#10-GTCGATAGC
#11-GTCATGCAC

S11b

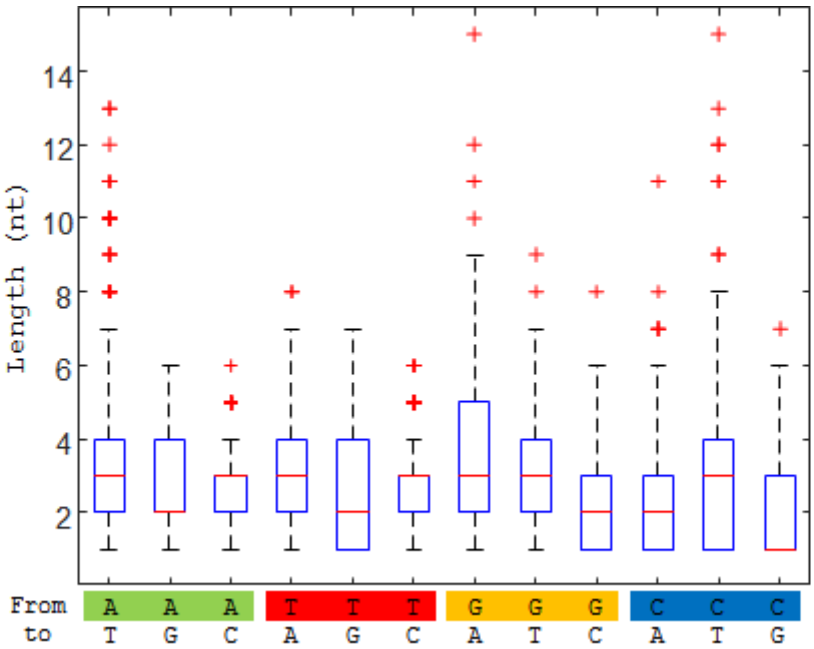

S11c

**Total reads**	**946872**
**Sequence with adaptor**	**20014**
**Perfect transition match**	**569**

Sequence	GAGACACTC	GAGTATAGC	GAGCATGAC	GACTATAGC	GACGCATC	GACACGAGC	GATCGCGAC	GATATGCTC	GATGCTAGC	GTCTGCATC	GTCGATAGC	GTCATGCAC
Sequence index	0	1	2	3	4	5	6	7	8	9	10	11
Filter 1,2,3 (# of transition, index)	252	166	89	86	106	66	74	175	51	112	47	57
Filter 4 (perfect match)	46	78	32	56	77	18	22	121	16	63	24	16

Supplementary Figure 11: In-depth analysis using Oxford Nanopore MinION sequencing. **a,** Box plot statistical information for the extension length distribution for each base transition for all perfectly matched (*in silico* filter 1, 2, 3, and 4) 12 oligonucleotides synthesized in multiplex. Each SEQ represents an individual subset/index and DNA oligonucleotide. **b,** Statistics for the extension length distribution for all possible transitions from the entire array with red pluses being statistical outliers. **c,** Raw sequencing data was filtered as per specified elsewhere for perfect matches containing all 8 base transitions for each oligonucleotide index. The breakdown for filtering from the raw sequencing data is included.

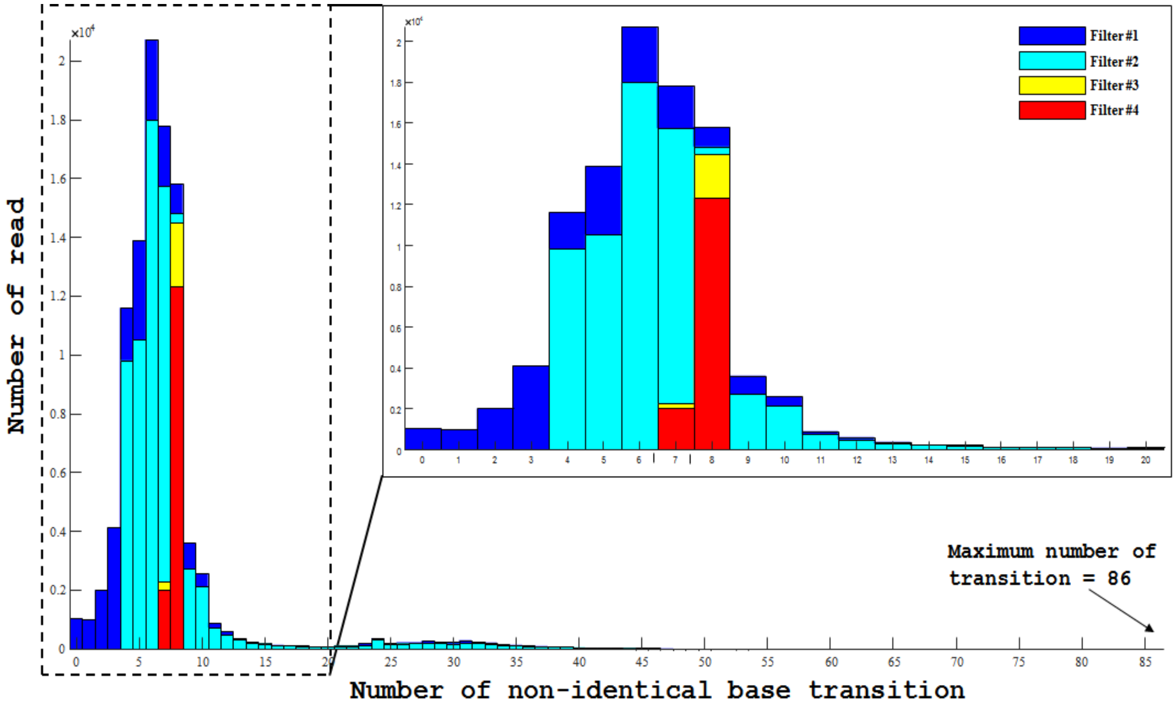

Supplementary Figure 12: A histogram indicating the distribution of non-identical base transitions for sequence reads after each filtering step from the Illumina MiSeq analysis of multiplex synthesis. **Filter #1** removes reads without sequencing adaptors from background noise (dark-blue bars). **Filter #2** removes sequencing reads without predetermined locational barcodes (sky-blue bars). Barcodes consisted of 3 base transitions. **Filter #3** removes sequencing reads that did not fall within the estimated number of total base transitions (yellow). In this case, 7-8 base transitions were expected. After this filter, stochastic estimation of the unknown sequences occurs. **Filter #4** removes sequencing reads that were not perfect matches to reference sequences. Only a small difference between **Filter #3** and **Filter #4** was observed, indicating that decoding data from the oligonucleotides synthesized in multiplex can be done without previously knowing any original sequence composition

References

1. Lee, H. H., Kalhor, R., Goela, N., Bolot, J. & Church, G. M. Terminator-free template-independent enzymatic DNA synthesis for digital information storage. *Nat. Commun.* **10**, 2383 (2019).
2. Rosen, S. & Howell, P. *Signals and Systems for Speech and Hearing*. (BRILL, 2011).
